# Data Visualization for Chronic Neurological and Mental Health Condition Self-management: Systematic Review of User Perspectives

**DOI:** 10.2196/25249

**Published:** 2022-04-28

**Authors:** Ashley Polhemus, Jan Novak, Shazmin Majid, Sara Simblett, Daniel Morris, Stuart Bruce, Patrick Burke, Marissa F Dockendorf, Gergely Temesi, Til Wykes

**Affiliations:** 1 Merck Research Labs, Information Technology Merck, Sharpe, & Dohme Zurich Switzerland; 2 Epidemiology, Biostatistics and Prevention Institute University of Zürich Zürich Switzerland; 3 Merck Research Labs, Information Technology Merck, Sharpe, & Dohme Prague Czech Republic; 4 School of Computer Science University of Nottingham Nottingham United Kingdom; 5 Institute of Psychiatry, Psychology and Neuroscience King's College London London United Kingdom; 6 RADAR-CNS Patient Advisory Board London United Kingdom; 7 Global Digital Analytics and Technologies Merck, Sharpe, & Dohme Kenilworth, NJ United States; 8 Merck Research Labs, Information Technology Merck, Sharpe, & Dohme Vienna Austria; 9 National Institute for Health Research Maudsley Biomedical Research Centre South London and Maudsley National Health Services Foundation Trust London United Kingdom

**Keywords:** digital health, remote measurement technology, neurology, mental health, data visualization, user-centered design

## Abstract

**Background:**

Remote measurement technologies (RMT) such as mobile health devices and apps are increasingly used by those living with chronic neurological and mental health conditions. RMT enables real-world data collection and regular feedback, providing users with insights about their own conditions. Data visualizations are an integral part of RMT, although little is known about visualization design preferences from the perspectives of those living with chronic conditions.

**Objective:**

The aim of this review was to explore the experiences and preferences of individuals with chronic neurological and mental health conditions on data visualizations derived from RMT to manage health.

**Methods:**

In this systematic review, we searched peer-reviewed literature and conference proceedings (PubMed, IEEE Xplore, EMBASE, Web of Science, Association for Computing Machinery Computer-Human Interface proceedings, and the Cochrane Library) for original papers published between January 2007 and September 2021 that reported perspectives on data visualization of people living with chronic neurological and mental health conditions. Two reviewers independently screened each abstract and full-text article, with disagreements resolved through discussion. Studies were critically appraised, and extracted data underwent thematic synthesis.

**Results:**

We identified 35 eligible publications from 31 studies representing 12 conditions. Coded data coalesced into 3 themes: desire for data visualization, impact of visualizations on condition management, and visualization design considerations. Data visualizations were viewed as an integral part of users’ experiences with RMT, impacting satisfaction and engagement. However, user preferences were diverse and often conflicting both between and within conditions.

**Conclusions:**

When used effectively, data visualizations are valuable, engaging components of RMT. They can provide structure and insight, allowing individuals to manage their own health more effectively. However, visualizations are not “one-size-fits-all,” and it is important to engage with potential users during visualization design to understand when, how, and with whom the visualizations will be used to manage health.

## Introduction

Despite widespread interest in the use of remote measurement technology (RMT) such as wearable devices and mobile apps in health care settings, the design and practical implementation of these technologies remain challenging. Designing RMT requires a careful balance between (often conflicting) requirements, which address technological, clinical, and regulatory specifications, individual users’ needs, and health-management goals. The result must be effective and engaging for users and capable of supplementing health management practices and promoting long-term adherence [1-3]. However, best practices and guidelines for RMT design are sparse and most designs rely heavily on designers’ interpretations or inferences of user preferences [4]. Emphasis on user engagement during RMT design has increased in recent years but is not yet a widespread practice [5].

Although determinants of engagement with RMT are diverse and condition-dependent, *access to and interaction with data* frequently emerge as a key factor influencing user motivation and satisfaction with RMT [6,7]. However, data access alone is insufficient to achieve goals like self-management. Data must be organized and presented in ways that meaningfully address questions of self-awareness and self-care [8]. To paraphrase Few [9], a good visualization must clearly indicate relationships, accurately represent the data, enable easy comparisons, clearly show scales and ordering of the data, and encourage people to use the presented information. This is not trivial, as perceptions of whether visualizations are accurate, clear, or easily interpretable are subject to personal mindset and circumstance [8]. Design of good visualizations for health management requires careful consideration of user perspectives and needs throughout the design process, ideally engaging these users through participatory design methods [10,11]. Recent research on health data visualization tends to focus on the needs of health care professionals, typically with regard to electronic medical records and other novel sources of big health data [12-14]. This paradigm is ripe for change. The abundance and accessibility of health data, driven in large part by RMT, provide individuals with unprecedented resources to aid in condition self-management.

Although theories and techniques exist to guide the visualization design process [15-17], these techniques require a fundamental understanding of the purposes for and contexts in which the desired visualizations will be used. Unfortunately, functional guidance describing service-user perspectives and preferences in RMT data visualization is scarce. A recent systematic review explored types of visualizations shown to patients in health research, predominantly in nondigital formats in primary care settings [18]. However, it did not discuss individuals’ perspectives on these visualizations. It is still unclear *which* data RMT users wish to access, *how* these data should be visualized, *for what purposes* these visualizations are used, and *which factors moderate* these preferences. The aim of this qualitative systematic review was to identify and synthesize existing studies that report the perspectives of individuals with neurological and mental health conditions on RMT data visualizations. Based on identified themes and gaps in the literature, we suggest both priorities for future research and design considerations for RMT visualizations, which could enrich current service-user engagement practices.

## Methods

### Study Scope and Research Question

This study adhered to PRISMA-P (Preferred Reporting Items for Systematic review and Meta-Analysis Protocols) guidelines for systematic review conduct and reporting [19,20]. We aimed to address the question, “What are the data visualization preferences and perceptions of people living with chronic neurological and mental health conditions when using RMT to manage health?” The project’s patient advisory board, which comprises patient advocates for depression, epilepsy, and multiple sclerosis, was consulted during the study design and data analysis.

### Identifying Relevant Studies

This protocol was registered on PROSPERO (International Prospective Register of Systematic Reviews, CRD42019139319) while the review was in its pilot phase [21]. We searched PubMed, IEEE Xplore, EMBASE, Web of Science, proceedings from the Association for Computing Machinery Conference on Human Factors in Computing Systems, and the Cochrane Library for original, peer-reviewed, or gray literature published in English between January 2007 and September 2021. Searches included combinations of terms such as mHealth, along with terms related to data visualization and neurological disease. The search strings are provided in Multimedia Appendix 1. Relevant papers were also identified from manual searches of included studies’ reference lists. Studies were screened in 2 stages: abstract screening and full-text review. Eligibility criteria and screening forms were piloted on a set of 50 abstracts and 15 full-text reviews, and criteria were clarified or amended as needed. Two reviewers (AP, JN, SM, or DM) independently screened each abstract. In the case of disagreement, the abstract automatically proceeded to the full-text stage. Two reviewers then independently assessed each full-text paper for eligibility, and disagreements were resolved through discussion. If no consensus could be reached, a third member of the review team reviewed the paper and made a final determination. Agreement between reviewer pairs was determined through Cohen kappa [22]. Deduplication, record management, and screening were conducted in CADIMA, an open-access systematic review software [23]. Data extraction and coding were conducted using custom forms developed in Microsoft Word (Multimedia Appendix 1).

### Eligibility Criteria

We included studies if they met the following criteria:

- All or part of a study population was living with a neurological or mental health condition
- Participants were ≥18 years of age
- RMT for laypeople to track, monitor, or manage their own health was investigated
- Results of any qualitative methods or integrated syntheses of mixed methods were reported
- Patient perspectives on visualizations of health or wellness data were reported

The following studies were ineligible for inclusion:

- Conditions that were not neurological in nature or not associated with mental health
- Perspectives on interface design, intervention design, or any component of RMT design unrelated to data visualization
- Visualizations limited to medication adherence or non–health-related data
- Perspectives of caregivers, health professionals, or others not living with a neurological or mental health condition

We adhered to Davis et al’s [24] definition of RMT, which includes “any technology that enables monitoring of a person’s health status through a remote interface,” which can then either be transmitted to a health care provider or as a tool for self-management of one’s health. We purposefully remained broad in our definition of the term “data visualization” because it is understood differently by different people. Therefore, we included any format through which RMT displayed data to service users. We defined chronic neurological or mental health conditions as any long-term, progressive, relapsing, or recurrent conditions related to mental health or dysfunction of the nervous system. Neurodegenerative diseases, depression, anxiety, or bipolar disorder, pain disorders, and sleep disorders, among others were eligible. Mental health conditions were also eligible if they were symptoms or comorbidities of a nonincluded condition. This heterogeneous set of conditions allowed us to identify themes that may be generalizable across conditions and others that are disease-specific.

### Data Extraction, Critical Appraisal, and Qualitative Synthesis

Two authors independently reread each included study and extracted quotes related to data visualization preferences. When available, screenshots of data visualizations were also extracted. To ensure that analysis remained grounded in the context of the original studies, data extraction forms included a detailed description of each study’s objectives and methods, and annotated PDFs were preserved. Studies were critically appraised with the Mixed Methods Appraisal Tool [25,26]. We then categorized studies as conceptually rich “key papers,” “satisfactory papers,” which are methodologically acceptable but provide only moderate value to the synthesis, and “fatally flawed papers,” which contain major methodological flaws [27,28]. We also noted “minimal impact papers,” which provided minimal contribution to the synthesis.

We employed Thomas and Harden’s [29] inductive approach to thematic synthesis. Following a reading of the extracted text and its context, 2 authors (AP and JN) independently coded data line by line, producing a draft coding frame. The coding frame was iteratively amended, refined, restructured and the data recoded until no additional codes or disagreements were identified, and categorized into “descriptive themes,” which described the structure and content of the codes. Analytical themes, which interpreted the coded data, were developed through iterative rereading and discussion of the codes and thematically organized data (Table 1). An experienced qualitative researcher (SS) oversaw and provided input on this process. Following thematic synthesis, we conducted sensitivity analyses to identify potential differences between conditions. We analyzed 4 subgroups (mental health, neurological conditions, sleep, and pain) that arose from the patient populations and lines of inquiry addressed by included studies. As a form of member-checking, we consulted members of the patient advisory board (authors PB and SB) who reviewed our analysis and interpretations for face validity within the context of their own experiences of RMT and condition self-management.

**Table 1:** Worked examples of the data synthesis process.

Extracted text	Coding	Descriptive theme	Analytical theme
*Participants reported that the mood monitoring surveys and associated graphical feedback were a reason to return to the app and that it increased self-awareness of how their mood fluctuated over time and in relation to use of the intervention content* [30].	Form: Graphical Self-awareness See progress	Increased self-awareness	Visualizations enable proactive self-management through improved self-awareness
*…It was really nice to look at the circle plot [the pie-chart]. When I’ve had a hard day, I looked back on the previous day, and saw a big yellow portion [Work and Education] and then it made sense to me why I felt bad today* [31].	Form: Graphical Identify patterns	Increased self-awareness	Visualizations enable proactive self-management through improved self-awareness

## Results

### Included studies

**Figure 1:**
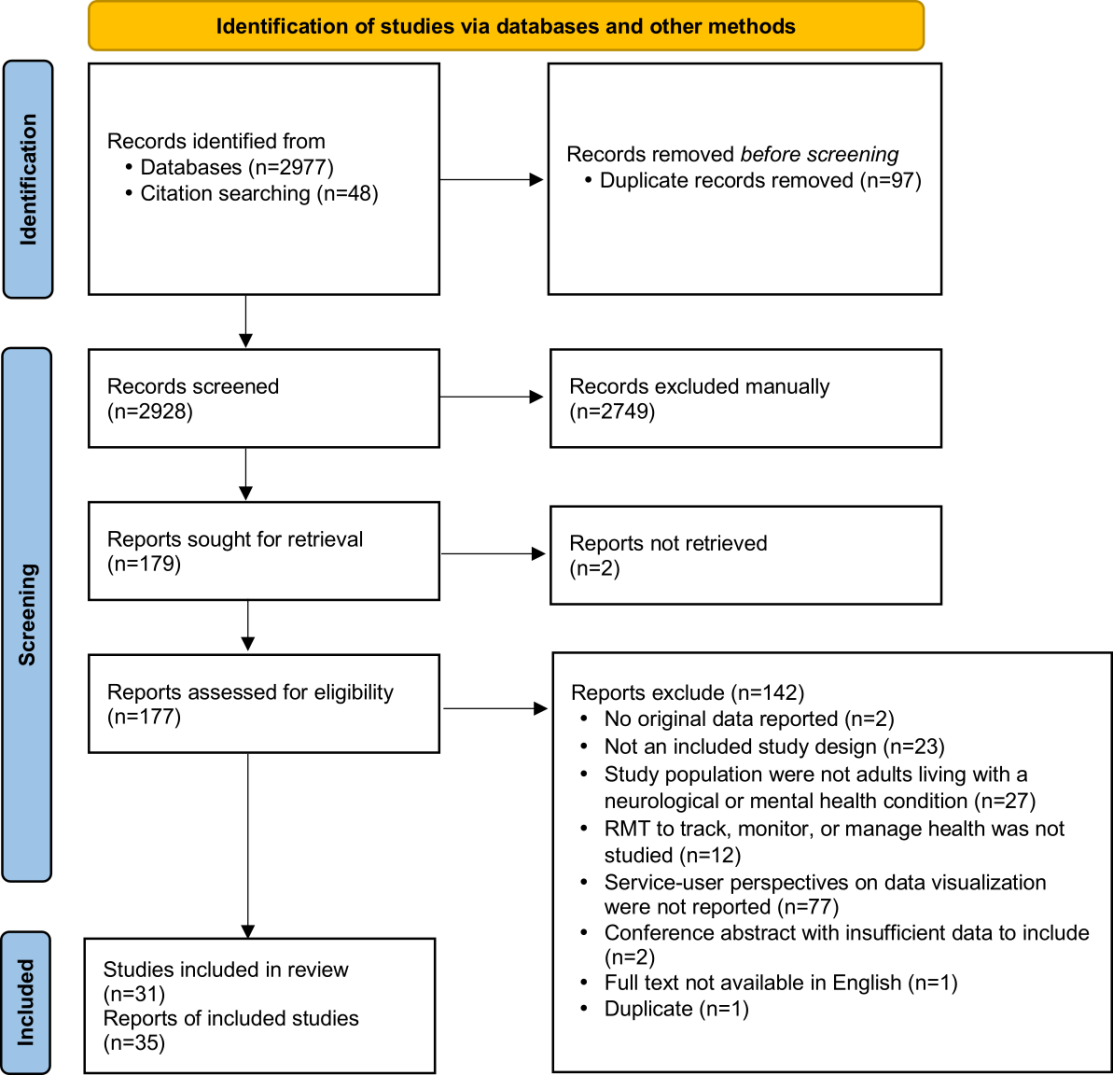
PRISMA (Preferred Reporting Items for Systematic review and Meta-Analysis) flow diagram of study screening and selection. RMT: remote measurement technology.

Searches returned 2928 unique records. Of these, 177 papers were included in full-text review and 35 were eligible for qualitative synthesis (Figure 1). Reviewer agreement was moderate during abstract screening (weighted κ=0.45) and substantial during full-text review (weighted κ=0.79) [22]. Our relatively low agreement during abstract screening was expected and mitigated by reviewing the full texts of all abstracts, which were judged eligible by at least one reviewer. Multiple papers were identified for 3 research projects: the SPIRIT study (n=3) [32-34], the MoodRhythm app (n=2) [35,36], and the MONARCA (MONitoring, treAtment and pRediCtion of bipolAr Disorder Episodes) project (n=2) [37,38]. For these projects, all identified papers were analyzed as 1 study. Thus, 31 unique studies were included. The characteristics of the included studies are summarized in Table 2 and provided in detail in Multimedia Appendix 2. Most studies (19/31, 61%) addressed mental health conditions. Only 2 studies specifically investigated user perspectives on data visualizations [39,40]. All others reported on data visualization preferences within the broader context of RMT design, evaluation, or usability. We categorized 9 publications as key papers, 16 as satisfactory papers, 6 as minimal impact, and 4 as fatally flawed. We identified 3 themes through content analysis: desire for data visualization, impact of visualizations on condition management, and visualization design considerations (Textbox 1). The final coding frame and illustrative quotes are provided in Multimedia Appendix 2.

**Table 2:** Characteristics of the included studies (N=31).

Characteristics			Values, n (%)^a^
**Condition**			
	Bipolar disorder	6 (19)	
	Depression	6 (19)	
	Sleep disorders	2 (6)	
	Schizophrenia	2 (6)	
	Anxiety	2 (6)	
	Pain disorders	2 (6)	
	Posttraumatic stress disorder	2 (6)	
	Mixed mental health conditions	6 (19)	
	Parkinson disease	2 (6)	
	Multiple sclerosis	3 (10)	
	Motor neuron disease	1 (3)	
	Epilepsy	1 (3)	
**Study design**			
	Qualitative feedback on RMT^b^ from a field study	15 (48)	
	Co-design of an RMT	10 (32)	
	Qualitative user acceptance testing of an RMT	11 (35)	
	Exploratory qualitative studies not associated with a specific RMT	10 (32)	
	Qualitative studies specifically on data visualization preferences	2 (6)	
**Qualitative methods**			
	Mixed methods	12 (39)	
	Interviews	17 (71)	
	Open-ended surveys	4 (13)	
	Focus groups	4 (23)	
	Other	3 (10)	

^a^As several projects included multiple conditions, study designs, or qualitative methods, the total exceeds 100%.

^b^RMT: remote measurement technology.

**Identified themes and subthemes.**

Theme 1. Desire for data visualization

Theme 2. Impact of visualizations on condition management

- Visualizations enable proactive self-management through improved self-awareness
- Visualizations enable more effective communication with care partners
- Visualizations drive engagement with remote measurement technology

Theme 3. Visualization design considerations

- Format
- Context and Annotation
- Customization
- Moderators of visualization design preferences

### Desire for Data Visualization

Users explicitly expressed a desire for data visualizations in 21 of the 31 included studies. Discussed or desired data included mood and disease-specific symptom scores, physical activity, sleep patterns and quality, and rhythm of daily activities. In 6 studies, RMT did not originally include visualizations of users’ historic data. Participants expressed their dissatisfaction, prompting authors to add visualizations to their designs [32-35,38,41,42].

### Impact of Visualizations on Condition Management

#### Visualizations Enable Proactive Self-management Through Improved Self-awareness

Participants reported turning to RMT and data visualization tools when they required external structure or organization to manage their conditions. This structure improved users’ recall of their own experiences, which were perceived as valuable owing to the difficulty of accurately reflecting on past symptoms [30,31,35,37,41,43]. Objective visualizations of past symptoms also afforded them a sense of validation, making current experiences feel more real or trustworthy [35,41,44]. This was especially important in bipolar disorder or schizophrenia, when symptoms could distort one’s perception of reality [35,41].

Graphs and charts enabled users to identify patterns [30,31,34-38,41-55] such as activities that prompted feelings of wellness [31,52,55] or triggered symptoms [31,35,41,46,56]. Thus, visualization of historic data heightened service users’ self-awareness, leading them to engage in more proactive self-management [31,35-37,41,46,50,53,55]. Once trends were identified, users could use their newly acquired knowledge to avoid or pre-empt triggers, reducing the severity of oncoming episodes [46]. This awareness gave users a sense of accountability and level of control over their conditions [46,50] and helped them use the management tools at their disposal more effectively [41,50,52]. Some saw timely access to data as essential to proactive self-management, especially when identifying recent triggers or activities that may have affected their current condition [35,36]. Over time and despite fluctuations, visualizations helped users objectively see their own progress, the magnitude of which they may not have fully appreciated otherwise [30,35,41,52,57]. Illustrative quotes are provided in Table 3.

**Table 3:** Illustrative quotes for visualizations improve self-awareness and enable proactive self-management.

Theme	Illustrative quotes
Provide structure and organization	…*Still, respondents reported turning to external tracking via paper or technology when they need extra support, for instance, when thoughts get “scrambled” or their mind feels too “full” - sensations that are especially common during [bipolar disorder] episodes* [43].
Improve recall of past experiences	…*What I saw [in the trial] is that it helped me keep on track. I try to keep track of the triggers [early warning signs], and my history - and in that way it has helped me enormously. Previously, I went into periods where I encountered random mood swings, up and down, and I did not have any history [data] to relate to, so it kind of surprised me. But now I can actually follow how I’m doing - also back in time - and what caused it. It has really been great, and I think I have been able to keep track of myself* [37] (bipolar disorder).
Validate current experiences	…*I am looking for confirmation that I had similar symptoms in the past, because sometimes due to the nature of bipolar I feel like I can't trust the emotions I have at any given moment (or their possible triggers) and it is a relief to know that these are patterns* [35] (bipolar disorder).
Increase self-awareness, identify patterns	…*I found the most valuable tool to analyze my activities. It provides an understanding of which activities helps me, and which gives problems that I need to be aware of—or completely avoid* [31] (depression).
Enable proactive self-management	…*A majority of respondents described this awareness as an opportunity to become proactive about their condition, helping them make adjustments to preempt mood episode triggers and maintain stability or at least avoid severe episodes. Survey respondents also stated that the feedback provided by tracking keeps them accountable to themselves and that they find visual forms of feedback particularly helpful for identifying personal behavioral and emotional patterns and motivating positive lifestyle choices* [43] (bipolar disorder).
See progress over time	…*I was AMAZED when I scrolled back through the Android weeks to see how much my mood has stabilized since I started [medication]. The weeks themselves weren't as meaningful as the pattern over time* [35] (bipolar disorder).

#### Visualizations Enable More Effective Communication With Care Partners

Participants also frequently reported using or desiring to use RMT data visualizations to communicate with health care professionals, caregivers, and others, regardless of whether the technology was designed for this purpose [39,41,43-46,50-55,58,59]. Eisner et al [41] described *“*participants theorized that having access to objective data representing their symptoms, particularly in the graph form, might enable a shared understanding of their experiences, both with the care team…and potentially with the general public.”

Participants in several studies reported using visualizations from their self-tracking apps to foster higher-quality dialogue with their care team [39,41,43,45,46,50-52,58,60]. Visualizations were advantageous because they improved recall of past symptoms and relayed clinically relevant patterns that users found difficult to describe [43,46,50,52]. Visual aids also helped patients make the most out of short and infrequent appointments, especially when current health status was not representative of the patient’s experiences over previous weeks or months [46,50,55]. Sometimes, these visualizations even served as the basis for defensible positions when broaching difficult topics with their care team [41,46]. For example, 1 participant who experienced difficulties getting a care team to take his concerns seriously stated, “If you were to answer the questions and go to the doctor and say ‘look, these are my results, you can see clearly there’s a change, and these are my experiences,’ that would be substantial evidence for the doctor to then sit up and take note” [41].

#### Visualizations Drive Engagement With RMT

Participants also described how visualizations made experiences with RMT more engaging [30,31,34,35,41,42,49,51]. Users with bipolar disorder and insomnia suggested that data access and visualization could entice users to engage more with the RMT, since data and insights could be perceived as rewards [35,49]. However, those with depression, Parkinson disease, and multiple sclerosis warned that inappropriately designed visualizations could be demoralizing [32,34,35,47,57,61], which could lead to disengagement. When visualizations were unavailable, users tended to find RMT unengaging and unmotivating. In 2 studies, the lack of personalized graphs in early prototypes was thought to contribute to study dropout [34,42]. Qualitative feedback in one of these studies, which focused on narcolepsy management, led the designers to conclude, “to keep patients motivated to use the tool over a longer period, a personal visualization of recorded data is required” [42].

### Visualization Design Considerations

Participants often reflected on aspects of visualization design, such as format, the need for contextual information, timeliness, and customization (Table 4). Health status, data literacy, and previous experience with RMT appeared to moderate individuals’ needs and design preferences.

**Table 4:** Illustrative quotes on visualization design considerations.

Theme	Illustrative quotes
Visualization design	…*Similar to other personal health systems that use fishes and flowers as metaphors, we were looking for an appropriate metaphor for bipolar disorder. Many attempts were tried, including using metaphors like a scale, an equalizer, a river, a volcano, a dart board, and a radar, but we always had the case that some patients preferred one visualization, and others hated it* [37] (bipolar disorder).
Context and annotation	…*I would like to click [points on the daily mood graph] and see what I did on this day, since I was so well* [31] (depression or anxiety disorder). *…I kinda wish I could put a little note in and be like, “This is why I put this number.” I think, yeah. I guess that’s actually - it would have been really nice to have some sort of journaly type feature where I could make notes like that* [34] (bipolar disorder).
Timeliness of data access	*Comments mostly differed regarding the frequency of (feedback via data visualizations), again with a great range from daily feedback to one response per month:* …*Yes, one feedback per month. Or maybe every two weeks. In the case of warning signals also more frequently.* …*Yes a short feedback every two days or every day.* …*Yes absolutely. Once a week would be good or every two weeks. Not every day though* [58] (bipolar disorder).
Customization	…*Survey respondents’ approaches for tracking multiple indicators vary. Participants were divided between keeping separate journals or tools, each dedicated to chronicling a particular indicator, or tracking all items with a single chart or application. Sometimes, elaborate tracking setups are reported as necessary to accommodate such tracking habits in ways technologies do not currently support* [46] (depression).

#### Format

Studies described a variety of data visualization formats, including line, bar, and pie charts [45,61], calendar views [31], scales [62], mood clouds [51], traffic lights [63], and overlays on maps [61]. Several studies reported that graphical representations were preferred by most participants [30,51,64], though personal preferences varied [37,40,49]. Some users preferred to aggregate multiple data streams in a single visualization [31,45], while others preferred simple, nongraphical formats such as scales or textual descriptions [61,62]. Participants suggested that images and color were powerful tools to make data visualizations more meaningful and engaging [31,35,45,51,61]. However, these tools could also trigger emotional responses that affected users’ self-image [31-33,35,37,41,47,49,61].

#### Context and Annotation

Users also discussed the importance of contextual information when interpreting visualized data [31,32,34,35,42,49,50,58]. They often wished to annotate their data, thus providing “internal” context in the form of notes alongside a numeric score [31,32,34,42,58]. When used in conjunction with a graphical visualization, these annotations could help users generate insights, which were not evident from numeric data alone. Such internal context was also seen as valuable when using visualizations to communicate with health care providers since it augmented users’ memories of past experiences [32,34,42,58]. Often, external context explaining symptom scores or condition-specific concepts were also required to help users interpret visualizations. This was especially important when apps were intended for independent use, outside a health care professional’s oversight [32,40,55]. Some participants wanted semipersonalized feedback based on their recent scores [34,35,49,50], which could be as simple as an occasional reassurance that the users’ data were normal [50] or as complex as personalized health management advice [34,42,46].

#### Timeliness

Six studies [35,36,45,55,58,63] discussed the time frame for data access. Participants valued regular data access because it provided them with confidence and insight as they worked to manage their conditions. Preferences varied, including real-time, daily, or monthly feedback. This variety was observed even within relatively uniform user populations. Several studies concluded that it was best to incorporate flexibility into their designs, enabling each user to choose the time frame and amount of data to visualize.

#### Customization

Participants often expressed conflicting visualization design preferences [31,38,45,46,48,49,51,61,62], and authors noted this as a design challenge. Unsurprisingly, the ability to customize data visualizations was highly desirable [30,32-34,45,46,51,64], and personalization was seen as key to increase long-term engagement with RMT [37]. This included the ability to select which data to visualize [32,34,37,42,46], how to visualize it [37,46,51], and how often or rapidly to access it [35,36,45,58,63]. Some users even wished to add their own personally relevant data streams to RMT visualizations [42]. Often, users desired flexibility to manipulate and compare data streams, allowing exploration of one’s own data and generation of insights [34,45,46].

### Moderators of User Needs and Design Preferences

#### Health Status

Participants indicated that visualizations must be meaningful and sensitive to users’ personal experiences of their health conditions and comorbidities. Otherwise, they may unintentionally provide negative feedback. For example, one study on people with Parkinson disease [61] visualized walking activity as progress across a map of France. However, slow progress was interpreted as negative feedback within the context of the users’ mobility impairments [61]. Participants with multiple sclerosis recommended that visualizations should be designed to emphasize an individual’s progress against their own goals rather than an uncontextualized or absolute score [47]. When disease progression is tracked over the long term, it should be visualized in a positive light and contextualized within the resources, management strategies, and self-care activities that individuals can continue to use to manage their conditions [57]. Use of bright color was frequently seen as desirable and engaging in day-to-day health management [31,35,51], but muted colors were perceived as preferable when someone was acutely depressed or struggling to maintain a routine, since harsh colors (ie, red) emphasized lack of progress [32,33,35,51,61]. For conditions that caused vision impairment, such as multiple sclerosis, legibility and color contrast were considered important for visualizations [52,57]. Visualizations must also account for possible changes in health status due to disease progression or episodic fluctuation, which may alter user needs or functional abilities [41,42,46,47,62].

#### Data Literacy and “Data People”

Participants often referred to themselves in terms of whether they were “data people” when describing their desired level of complexity in visualizations. Data people tended to want more control over their visualizations to allow self-exploration of their symptoms and trends [45,46]. However, those who did not identify as data people preferred simpler visualizations. These individuals also tended to question the accuracy of purely numeric information [40,41]. This further highlights the need for flexibility, allowing users to choose how and how much data are displayed on a single visualization [64].

#### Experience With Self-monitoring

Some users described that their visualization preferences could evolve when self-monitoring was practiced regularly [34,35,40,46]. Users described developing their skills with just a few variables at first and then moving on to complex customized visualizations with many data streams [34,35,40,46]. Once they were accustomed to tracking many variables, self-monitoring became a mental task that no longer required RMT or visualizations. However, users reported returning to external tracking methods during acute phases or when something disrupted their routines. Those who had extensive experience with RMT also tended to request more customizable features such as selecting data streams to display or requesting personalized advice [34,46]. If customization is not available, there is a risk of disengagement with the RMT as users’ management practices evolve over time [46].

## Discussion

### Principal Findings

**Figure 2:**
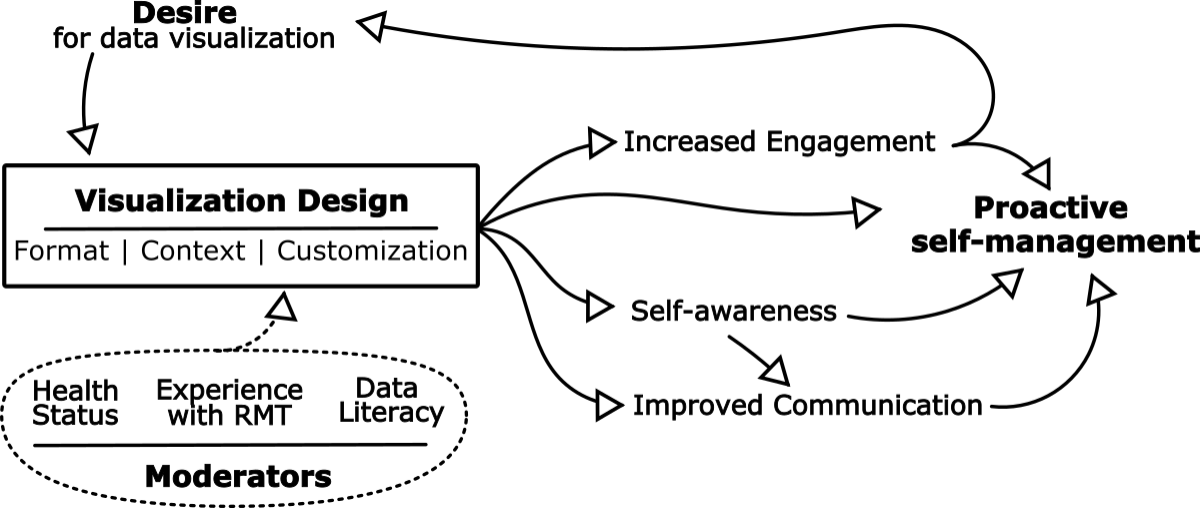
Data visualization as an integral personal component of health management. RMT: remote measurement technology.

When designed appropriately, data visualizations are perceived as valuable, engaging components of RMT. They can be validating and empowering, allowing users to become more active, responsible participants in their own care. Individual experiences of health conditions were perceived as highly personal, leading to requests for contextual aids, flexibility, and personalization. Factors such as health status, data literacy, and past experiences affected user needs, thereby moderating design preferences. Figure 2 describes the themes and relationships identified in this study.

Ample literature discusses scientifically derived best practices for app interface design, engagement strategies, and intervention design in various medical conditions and contexts [65-67]. Many of these modern practices emphasize user engagement, co-design, and human-centric design methods with an emphasis on scientific rigor [5,66,68]. In fact, many included studies reported on app development processes that adhered to these principles. Unfortunately, specific literature on visualization design within the context of RMT-enabled health management is sparse. This review aimed to address this gap. Based on our synthesis alone, we cannot suggest concrete examples of good design or universal design preferences. On the contrary, this review highlighted the diversity of preferences both across and within conditions and study samples. However, with this in mind, we pose several recommendations for RMT visualization design.

First, visualizations of personal data were consistently considered valuable, engaging, and even necessary components of RMT. When designed appropriately, they can be an integral part of condition self-management. Therefore, incorporation of data visualizations should be considered when designing RMT. However, designs should be sensitive to the experiences and health statuses of their target audiences. Participants were regularly concerned that visualizations that inadvertently emphasized slow progress, poor health, or unattained goals could prompt disengagement with RMT during periods of relapse or perceptible disease progression [32-34,42]. These warnings are consistent with the findings of Lee et al [69] who suggest that negative emotions and subsequent reductions in health self-efficacy reduce health information–seeking behavior [69].

Designs should be informed by a deep understanding of the service users’ needs and experiences. For example, study participants with rapid-cycling bipolar disorder suggested that tracking apps and visualizations were not useful to them since most apps allowed only 1 data entry per day [42,46]. Such mismatches between user needs and design features are avoidable if sufficient time is taken *early in the design process* to truly empathize with service users [5]. Designers should work with members of the target audience to understand their lived experiences, needs, and challenges. It is critical to engage with potential users during the design phase to understand *when, how, and with whom* data visualizations will be used. Fit-for-purpose visualizations can then be designed with these factors in mind.

Included studies repeatedly suggested that visualizations are not “one-size-fits-all.” Symptoms, triggers, contextual factors, and tracking needs differ from person to person, context to context, and timepoint to timepoint. As such, individual data visualization preferences are highly variable and often dynamic. This is consistent with Paterson’s [70] “shifting perspectives model of chronic illness,” which describes how individuals’ perceptions of their conditions, symptoms, and health fluctuate between predominant feelings of wellness and illness over time. Sometimes, individuals may predominantly self-identify as well. In a health-tracking context, this may lead service users to track symptoms less frequently or focus on wellness data (eg, physical activity, sleep) [31]. During such times, reminders of feelings of illness prompted by symptom tracking may even be detrimental to the service user’s self-image. However, when feelings of control over health status is threatened, such as during a relapse, individuals tend to shift their perspectives and more readily identify as ill [70]. During these times, individuals may be more inclined to self-track their symptoms to identify trends and regain control [46,50]. Owing to this interpersonal and intrapersonal variability, flexible design is highly desirable when it is feasible to implement. Designers should remain mindful of sources of heterogeneity in their target audience’s needs and subsequently identify RMT features that are most likely to require flexibility or personalization.

We also identified areas where tensions may emerge between user preferences and those of other stakeholders. Although this study focused on RMT designed for individual use, participants, especially those with mental health conditions, perceived data visualizations as tools to help communicate experiences to their care partners. However, clinicians often find it difficult to interpret and manage the visualizations derived from RMT [71], especially if visualizations are not standardized across the various apps used by patients. If visualizations are to be used as communication tools, they must be interpretable, meaningful, and actionable by those involved in patient care [72]. Otherwise, confusion or even miscommunication may ensue, potentially wasting valuable face-to-face time during short appointments with health care professionals [73]. Therefore, engagement with other possible users such as caregivers or health care professionals may also be valuable during visualization design.

### Limitations

As with all qualitative metasyntheses, the themes identified here are influenced by the topics, context, and limitations of the included studies. All results reported here must be interpreted as third-order constructs [74] or interpretations of interpretations of an individual’s original feedback. Although this is the aim of a meta-synthesis, it does pose limitations to remaining grounded in the authors’ and participants’ original meanings and contexts. As reviewers, we attempted to minimize our assumptions during coding and synthesis by continually questioning each other’s interpretations of the data. However, we acknowledge that is impossible to fully isolate our synthesis from our own experiences and worldviews. Study methodology was heterogeneous and the authors’ place in the data was not always clear. Most comments on visualizations were brief and conducted as part of a larger studies, yielding relatively shallow data. Few studies compared preferences between multiple visualization designs, limiting our ability to compare the merits or relative preferences between design options. Most studies used user experience and co-design methods, which generally did not meet traditional expectations of qualitative research rigor. Although we opted for broad search strategies and sensitive review methods to identify all relevant studies, data visualization was almost always a secondary topic in the included papers and was not always clearly discussed. It is therefore a possibility that our strategies did not identify all eligible papers. Those papers that were included predominantly reflected the perspectives of individuals with mental health conditions, and it is unclear how these results may translate to other populations.

### Future Work

Few studies directly addressed research questions related to data visualization preferences. Rather, data visualization was usually approached as peripheral piece of a larger project. Therefore, this review should serve as a starting point for targeted work on health data visualization design. Additional qualitative research on service-user data visualization preferences is warranted, especially within the context of neurological condition self-management. This research should involve individuals directly through interviews, focus groups, or other qualitative methods and should focus on generating “thick” qualitative data that describe more specific uses and contexts. This work should verify and build upon the concepts developed here. Ideally, this work should span multiple conditions and generate diverse perspectives. These perspectives should then be used to generate more detailed design considerations and recommendations for RMT visualizations.

### Conclusions

When designed appropriately, data visualizations are valuable engaging components of RMT. They can provide structure and insight, allowing individuals, especially those with mental health conditions, to manage their own health more effectively. However, visualizations are not “one-size-fits-all,” and it is important to engage with potential users during visualization design to understand when, how, and with whom visualizations will be used to manage health. The considerations presented here should serve as the basis for future designs and discussions to ensure that visualizations address users’ needs and preferences.

## Appendix

Multimedia Appendix 1 Search strategy and data extraction form.

Multimedia Appendix 2 Characteristics of the included studies, critical appraisal, coding, and illustrative quotes.

## Abbreviations

EFPIA: European Federation of Pharmaceutical Industries and Associations
MONARCA: MONitoring, treAtment and pRediCtion of bipolAr Disorder Episodes
NHS: National Health Service
NIHR: National Institute for Health Research
PRISMA-P: Preferred Reporting Items for Systematic review and Meta-Analysis Protocols
PROSPERO: International Prospective Register of Systematic Reviews
RADAR-CNS: Remote Assessment of Disease and Relapse-Central Nervous System
RMT: remote measurement technology
