# Supplementary material for: Data Visualization for Chronic Neurological and Mental Health Condition Self-management: Systematic Review of User Perspectives
Source: JMIR Ment Health. 2022 Apr 28;9(4):e25249. doi: 10.2196/25249 (PMC9100378; doi:10.2196/25249)
Supplement: Multimedia Appendix 1 [file mental_v9i4e25249_app1.pdf]

Multimedia Appendix 1: Search strategy and data extraction form

Databases:
Pubmed, IEEE Xplore, EMBASE, Web of Science, ACM CHI, Cochrane Library

Date:
January 2007 to September 2021

Language:
English

Search strings:
PubMed (https://pubmed.ncbi.nlm.nih.gov/)

(eHealth OR mHealth OR "digital health" OR "remote patient monitoring" OR "remote monitoring technology" OR "remote measurement technology" OR telehealth OR telemonitoring OR application OR wearable) AND (patient AND (data OR symptom*) AND (visual OR visualiz* OR visualis* OR present* OR graph*) AND (prefer* OR perception OR usability OR design OR acceptability)) AND ("central nervous system" OR "nervous system" OR psychiat* OR neurolog* OR neurodegen* OR "mental health")

IEEE Xplore (https://ieeexplore.ieee.org/Xplore/home.jsp)

((((("Abstract":ehealth OR mhealth OR "digital health'" OR "remote patient monitoring" OR "remote monitoring technology" OR "remote measurement technology" OR telehealth OR telemonitoring OR application OR wearable) AND "Abstract":visual OR visualiz* OR visualis* OR present* OR graph*) AND "Abstract":"central nervous system" OR "nervous system" OR psychiatric OR psychiatry OR neurology OR neurological OR neurodegeneration OR "mental health") AND "Abstract":Preference OR preferences OR preferred OR perception OR usability OR design OR acceptability) AND "Abstract":patient AND (data OR symptom OR symptoms))

EMBASE* (https://www.embase.com/)

(ehealth OR mhealth OR 'digital health' OR 'remote patient monitoring' OR 'remote monitoring technology' OR 'remote measurement technology' OR telehealth OR telemonitoring OR application OR wearable) AND patient AND (data OR symptom*) AND (visual OR visualiz* OR visualis* OR present* OR graph*) AND (prefer* OR perception OR usability OR design OR acceptability) AND ('central nervous system' OR 'nervous system' OR psychiat* OR neurolog* OR neurodegen* OR 'mental health') AND [2007-2020]/py AND [humans]/lim AND [english]/lim AND 'article'/it

Web of Science* (https://login.webofknowledge.com/)

(ALL = ((ehealth OR mhealth OR "digital health'" OR "remote patient monitoring" OR "remote monitoring technology" OR "remote measurement technology" OR telehealth OR telemonitoring OR application OR wearable) AND patient* AND (data OR symptom*) AND (visual OR visualiz* OR visualis* OR present* OR graph*) AND (prefer* OR perception OR usability OR design OR acceptability) AND ("central nervous system" OR "nervous system'"OR psychiat* OR neurolog* OR neurodegen* OR "mental health"))) AND LANGUAGE: (English) AND DOCUMENT TYPES: (Article) Indexes=SCI-EXPANDED, SSCI, A&HCI, ESCI Timespan=2007-2020

ACM* (https://dl.acm.org/)

(eHealth OR mHealth OR "digital health" OR "remote patient monitoring" OR "remote monitoring technology" OR "remote measurement technology" OR telehealth OR telemonitoring OR application OR wearable) AND (patient AND (data OR symptom*) AND (visual OR visualiz* OR visualis* OR present* OR graph*) AND (prefer* OR perception OR usability OR design OR acceptability)) AND ("central nervous system" OR "nervous system" OR psychiat* OR neurolog* OR neurodegen* OR "mental health")

Cochrane Library (https://www.cochranelibrary.com/)

(eHealth OR mHealth OR "digital health" OR "remote patient monitoring" OR "remote monitoring technology" OR "remote measurement technology" OR telehealth OR telemonitoring OR application OR wearable) AND (patient AND (data OR symptom*) AND (visual OR visualiz* OR visualis* OR present* OR graph*) AND (prefer* OR perception OR usability OR design OR acceptability)) AND ("central nervous system" OR "nervous system" OR psychiat* OR neurolog* OR neurodegen* OR "mental health")

Data extraction form

RefID:				
**Population:**				**Description/Purpose of App/Study:**
**Type of Study**			**N**	
	Exploratory qual study (no spec. app)			
	User-centric design/co-design			
	App usability (lab-based)			**Data Visualized:**
	Field Test			**User Acceptance Scores:**
	Other			**Study Risk of Bias/Quality Assessment:**
**Qualitative data:**				**Link to annotated PDF:**
**Mixed methods?:**				**Other notes:**

screenshots	Background about screenshots

Page	Data Visualization: Extracted Data/Quotes for coding(context in italics)	Codes
