# Supplementary material for: Data Visualization for Chronic Neurological and Mental Health Condition Self-management: Systematic Review of User Perspectives
Source: JMIR Ment Health. 2022 Apr 28;9(4):e25249. doi: 10.2196/25249 (PMC9100378; doi:10.2196/25249)
Supplement: Multimedia Appendix 2 [file mental_v9i4e25249_app2.pdf]

Multimedia appendix 2: Included studies & data analysis

Table of Contents

Characteristics and Critical Appraisal of Included Studies 2

Table S1: Characteristics of included studies 2

Table S2: Critical appraisal and impact assessment of included qualitative studies 5

Table S3: Critical appraisal and impact assessment of included mixed-method studies 6

Coding Frame 7

Table S4: Coding frame developed for this study. 7

Illustrative Quotes 10

Table S5: Theme 1 - Desire for Data Visualization. 10

Table S6: Theme 2 - Impact of visualizations on condition management 10

Table S7: Sub Theme 2.1 - Visualizations improve self-awareness and enable proactive self-management. 11

Table S8: Sub Theme 2.2 - Visualizations enable more effective communication with care partners. 12

Table S9: Sub Theme 2.3 - Visualizations drive engagement with RMT. 13

Table S10: Theme 3 - Visualizations as data reporting mechanisms 13

Table S11: Theme 4 – Visualization design considerations. 14

Table S12: Sub theme 4.4 – Moderators of user needs and design preferences. 15

Characteristics and critical appraisal of included studies

Table S1: Characteristics of included studies

Study	Year	Condition	Description	Qualitative methods (n)	Data visualized
**Adams et al [40]**	2017	Chronic pain	Development of a user interface and application for reporting pain data through visuals and images	Co-design sessions (n=unclear)<br>In-lab usability study (n=10)<br>Field test & semi-structured interviews (n=12)	Pain severity
**Anderson et al [45]**	2016	Mixed mental health conditions	Exploratory study on use of mobile health apps to facilitate self-care. No specific RMT was studied.	Semi-structured interviews (n=22)	NA
**Ando et al [44]**	2019	Motor neurone disease	Explore service users' experiences with a telehealth and telemonitoring system (Careportal)	In-lab usability study (n=7)<br>Field test & semi-structured interviews (n=7, with carers' assistance due to condition severity)	Condition-related physiological data, often respiratory
**Bardram et al [37,38]**	2012, 2013	Bipolar disorder	Design and testing of a mobile health system (MONARCA) for symptom monitoring and management	Co-design sessions (n=7, plus 3 HCPs)<br>Field test & semi-structured interviews (n=12)	Mood, sleep, activity level, 'warning signs,' stress, medication adherence
**Bauer et al [32-34]**	2017-2019	Depression, PTSD, bipolar disorder	Mobile health system (SPIRIT application) for primary care-based mental health services in rural areas	User-centric design (n= 1+)<br>Lab-based usability study (n=16)<br>Field test & semi-structured interviews (n=10)	Anxiety and depression scores (GAD-7 and PHQ-9)
**Buitenweg et al [62]**	2019	Mixed severe mental health conditions	Development of a quality of life assessment application (QoL-ME) which minimized dependency on language for health tracking	User-centric design (n= 35)<br>Lab-based usability study (n=24)	Quality of life scores
**Daus et al [58]**	2018	Bipolar disorder	Exploratory mixed-methods study to understand service user perspectives on condition self-management with mobile applications	Semi-structured interviews (n=15)	NA
**Eisner et al [41]**	2019	Schizophrenia	Development and testing of the ExPRESS mobile application for monitoring symptoms and early signs of psychosis relapse	Field test & semi-structured interviews (n=18)	Occurrence of delusions and hallucinations
**Elsborg et al [54]**	2020	Anxiety	Explore patient experience and acceptability of using physiological data visualization to support self-reflection and manage anxiety	Field test & semi-structured interviews (n=2)	Galvanic skin response
**Erten-Uyumaz et al [49]**	2019	Insomnia	Testing of a negation-based sleep scheduler app for treating insomnia	In-lab usability study (n=8)	Sleep duration, sleep efficiency, time in bed, naps
**Forchuk et al [48]**	2015	Mixed mood or psychotic conditions	Development and testing of an electronic personal medical record system (Lawson SMART Record)	Field test (n=394) and focus groups (n=95)	Various medical data and symptom scores
**Fuller-Tyszkiewicz et al [30]**	2018	Depression	User experience evaluation of BlueWatch, an app-based intervention and monitoring system for depression	In-lab usability study (total: n=5 people with depression plus 10 HCPs)	Mood scores, daily activities

**Garzo et al [61]**	2018	Parkinson's disease	User experience evaluation of an app-based gait training system (BeatHealth)	In-lab usability study (n=12)<br>Field test & semi-structured interviews (n=37)	Distance walked, gait speed, training summary
**Giunti et al [47]**	2018	Multiple sclerosis	Exploratory study on MS-specific service user needs for mHealth-based physical activity tracking	Focus groups (n= 10)<br>Semi-structured interviews (n=2)<br>*Parallel study with HCPs:*<br>Focus groups (n= 8)<br>Semi-structured interviews (n=4)	NA
**Herbst et al [56]**	2020	PTSD, addiction	Evaluation of an app-based cognitive behavioral therapy for smoking cessation in people with PTSD	Field test & semi-structured interviews (n=17)	Use of tobacco products
**Hoffman et al [43]**	2019	Mixed mental health conditions (depression, anxiety, sleep, others)	Feasibility study of a smartphone app to augment primary care mental health services	Exploratory qualitative study: (n=Unclear)<br>Field test & qualitative survey (n=54)	Unclear
**Jonassaint et al [39]**	2018	Chronic pain	Feasibility study of an app (Painimation) for communicating and assessing chronic pain through visuals and animations	Co-design and semi-structured interviews (n=16)<br>Field test and semi-structured interviews (n=170)	Pain type, intensity, and location
**Matthews et al [35,36]**	2015, 2016	Bipolar disorder	Design and testing of an application (Moodrhythm) for symptom tracking and daily rhythm regulation	Co-design sessions (n=9)<br>Field test & semi-structured interviews (n=9)	Mood, daily activities, energy level, sleep
**McClelland et al [51]**	2018	Mixed mental health conditions	Development and testing of a mobile application for symptom tracking and condition management	Exploratory focus groups & Co-design sessions (n=10, plus 8 HCPs)	Mood, symptom scores
**Meyer et al [24]**	2018	Schizophrenia	Development and testing of a mobile health platform (Sleepsight) and consumer-grade fitness trackers for sleep and restful activity tracking	Field test & survey, partially open-ended (n=15)	Sleep, activity level
**Murnane et al [46]**	2016	Bipolar disorder, depression	Exploratory study on service user experiences and preferences for technology-based health monitoring	Open-ended survey (n=552)	NA
**Ospina-Pinillos et al [63]**	2019	Mixed mental health conditions	Testing and cultural adaptation of the Mental Health eClinic application in Spanish speakers	Co-design sessions (n=10, plus 7 HCPs)<br>In-lab usability study (n=7, plus 5 HCPs and 3 caregivers)	Physical and mental health self-assessments
**Proudfoot et al [64]**	2010	Anxiety, depression	Exploratory study on service user perspectives on mobile health technologies to manage depression, anxiety, and stress. No specific RMT was studied.	Focus groups (n=47)<br>Semi-structured interviews (n=20)<br>Survey, partially open-ended (n=525)	NA
**Quaedackers et al [42]**	2020	Narcolepsy	Design and testing of an application (Narcolepsy Monitor) for long-term symptom monitoring	Field test & semi-structured interviews (n=7)	Set of 18 symptom scores

**Rohani et al [31]**	2019	Depression	Development and testing of an app-based behavioral activation intervention for people with depression	Co-design sessions (n=2)<br>Field test & open-ended survey questions (n=7)	Mood, daily activities, Pleasure during activities
**Ruzic et al [55]**	2017	Multiple sclerosis	Identify health self-management needs for mHealth apps for individuals aging with MS	Focus groups (n=8)	NA
**Saunders et al [50]**	2017	Bipolar disorder	Study on service user perspectives on mobile health technologies to manage bipolar disorder following an interventional study with the MoodZoom self-management system.	Semi-structured interviews (n=21)	Mood, daily activities, symptom scores
**Schleimer et al [57]**	2020	Multiple sclerosis	Design and development of Open MS BioScreen platform, a management and tracking tool for MS.	Co-design sessions, interviews, and focus groups (n=74 patients plus 8 HCPs and 4 advocacy groups)	Disease severity score, long-term trajectory
**Van Tiem et al [53]**	2020	Depression	Evaluation of a text messaging intervention for depression self-management	Field test & semi-structured interviews (n=21)	Trajectories of symptom severity, function, and self-management strategies
**Wannheden et al [52]**	2020	Parkinson's disease	Explore the expectations and desired eHealth functionalities of people with Parkinson's and healthcare professionals with respect to co-care	Focus groups (n=7)	NA
**Yoo et al [59]**	2020	Epilepsy	Design and evaluate Brain4U, a seizure tracking system for people with epilepsy	In-lab usability study (n=3 patients plus 5 caregivers)	Seizures, triggers

Critical appraisal was conducted using the mixed-methods appraisal tool (MMAT), and studies were appraised as qualitative (n= 15) or mixed-method (n=14) studies [25,26]. The MMAT does not provide a total quality score. Rather, it prompts the user to assess a study's quality according to a series of questions to which the review can answer "Yes," "No," or "Can't Tell" (Noted as "Y," "N," and "?," respectively, in Tables S2 and S3). We then categorized studies as 'key papers' which are conceptually rich with an important contribution to the synthesis, 'satisfactory papers' which are methodologically acceptable but provide only moderate value or contribution to the synthesis, and 'fatally flawed papers' which contain major methodological flaws [27,28]. In addition, we note 'minimal impact papers' in which relevant data was sparse and which provided minimal contribution to the synthesis.

Table S2: Critical appraisal and impact assessment of included qualitative studies

Study	Year	Are there clear research questions?	Do the collected data allow to address the research questions?	Is the qualitative approach appropriate to answer the research question?	Are the qualitative data collection methods adequate to address the research question?	Are the findings adequately derived from the data?	Is the interpretation of results sufficiently substantiated by data?	Is there coherence between qualitative data sources, collection, analysis and interpretation?	Overall quality and contribution to the synthesis
**Anderson et al [45]**	2016	Y	Y	Y	Y	Y	Y	Y	Satisfactory
**Ando et al [44]**	2019	Y	Y	Y	Y	?	Y	Y	Minimal impact
**Bauer et al [32-34]**	2017-2019	Y	Y	Y	Y	Y	Y	Y	Satisfactory
**Buitenweg et al [62]**	2019	?	?	?	?	Y	Y	Y	Minimal impact
**Daus et al [58]**	2018	Y	Y	Y	Y	Y	Y	Y	Satisfactory
**Eisner et al [41]**	2019	Y	Y	Y	Y	Y	Y	Y	Key paper
**Elsborg et al [54]**	2020	Y	N	Y	N	N	N	N	Fatally flawed
**Erten-Uyumaz et al [49]**	2019	Y	Y	Y	Y	Y	Y	Y	Satisfactory
**Herbst et al [56]**	2020	Y	Y	Y	Y	Y	Y	Y	Satisfactory
**Matthews et al [35,36]**	2015, 2016	Y	Y	Y	Y	Y	Y	Y	Key paper
**McClelland et al [51]**	2018	Y	Y	Y	Y	Y	Y	Y	Satisfactory
**Murnane et al [46]**	2016	Y	Y	Y	Y	Y	Y	Y	Key paper
**Ospina-Pinillos et al [63]**	2019	Y	Y	Y	Y	Y	Y	Y	Minimal impact
**Ruzic et al [55]**	2017	Y	Y	Y	Y	Y	Y	Y	Key paper
**Saunders et al [50]**	2017	Y	Y	Y	Y	Y	Y	Y	Satisfactory
**Schleimer et al [57]**	2020	Y	Y	Y	Y	Y	Y	Y	Key Paper
**Van Tiem et al [53]**	2020	Y	Y	Y	Y	Y	Y	Y	Satisfactory
**Wannheden et al [52]**	2020	Y	Y	Y	Y	Y	Y	Y	Satisfactory

Table S3: Critical appraisal and impact assessment of included mixed-method studies

STUDY	YEAR	ARE THERE CLEAR RESEARCHQUESTIONS?	DO THE COLLECTED DATA ALLOW TO ADDRESS THE RESEARCH QUESTIONS?	IS THERE AN ADEQUATE RATIONALE FOR USING A MIXED METHODS DESIGN TO ADDRESS THE RESEARCH QUESTION?	ARE THE DIFFERENT COMPONENTS OF THE STUDY EFFECTIVELY INTEGRATED TO ANSWER THE RESEARCH QUESTION?	ARE THE OUTPUTS OF THE INTEGRATION OF QUALITATIVE AND QUANTITATIVE COMPONENTS ADEQUATELY INTERPRETED?	ARE DIVERGENCES AND INCONSISTENCIES BETWEEN QUANTITATIVE AND QUALITATIVE RESULTS ADEQUATELY ADDRESSED?	DO THE DIFFERENT COMPONENTS OF THE STUDY ADHERE TO THE QUALITY CRITERIA OF EACH TRADITION OF THE METHODS INVOLVED?	CONTRIBUTION TO THE SYNTHESIS
**Adams et al [40]**	2017	Y	Y	Y	Y	Y	?	?	Key paper
**Bardram et al [37,38]**	2012, 2013	Y	Y	Y	Y	Y	Y	Y	Key paper
**Forchuk et al [48]**	2015	Y	Y	Y	Y	Y	?	Y	Satisfactory
**Fuller-Tyszkiewicz et al [30]**	2018	Y	Y	Y	Y	Y	?	Y	Minimal impact
**Garzo et al [61]**	2018	Y	Y	Y	Y	Y	?	Y	Satisfactory
**Giunti et al [47]**	2018	Y	Y	Y	Y	N	N	?	Minimal impact
**Hoffman et al [43]**	2019	Y	Y	Y	Y	Y	?	N	Fatally flawed
**Jonassaint et al [39]**	2018	Y	Y	Y	Y	Y	Y	?	Minimal impact
**Meyer et al [24]**	2018	Y	Y	Y	N	N	?	N	Fatally flawed
**Proudfoot et al [64]**	2010	Y	Y	Y	Y	Y	Y	Y	Satisfactory
**Quaedackers et al [42]**	2020	Y	Y	Y	Y	Y	?	Y	Satisfactory
**Rohani et al [31]**	2019	Y	Y	Y	Y	Y	?	?	Satisfactory
**Yoo et al [59]**	2020	Y	Y	Y	N	?	?	?	Fatally Flawed

Coding frame

Table S4: Coding frame developed for this study. Codes were identified, organized, and grouped into descriptive themes (ie, “Visualization Design”, shown in gray), primary codes (ie, “Format”, listed by row), and sub codes (ie, “Numbers/text,” “Graphical”, shown as bullet points under primary codes).

Themes, codes & sub codes	Code definition
**Desire for visualization**	
**Desire for data visualization**<br>• Dissatisfaction with lack of visualization	Indication of a desire (or lack thereof) for visualizations of data derived from RMT, even when not originally provided in an RMT
**Visualization design**	
**Format**<br>• Numbers/text<br>• Graphical<br>• Non graphical<br>• Animation<br>• Images	Description of the form of the data visualization, either provided by the authors or desired/described by participants. Sub codes were used to describe common types of formats, such as line graphs (Graphical), numeric displays (Numbers/text), etc.
**Selecting, manipulating, comparing data streams**	Any discussion of selecting (e.g., choosing which data to visualize), manipulating (e.g., zooming in, analyzing or filtering), or comparing data streams through or within visualizations
**Context & annotation**<br>• Internal<br>• External	Any discussion related to contextual information required to interpret and use the visualization. Context could be internal (ie, self-reported notes to explain a specific score), or external (information provided by the app to help users interpret and act on the visualized data).
**Simplicity vs. Complexity**	Any discussion regarding service users’ preference on how simple or complex a visualization should ideally be. This code included preferences, general comments, and tensions relating to simplicity and complexity.
**Image, shape, & color**<br>• Convey meaning<br>• Cause feelings	Any discussion regarding ways in which image, shape, and color affected users’ experiences or preferences of a visualization. Often, color “conveyed meaning,” (ie, categorization of data), or “caused feelings” in a positive or negative manner.
**Timeliness of data access and visualization**	
**Timeliness**<br>• Real time<br>• Occasional<br>• Variable	Any description of how often or how quickly individuals preferred to access and visualize their data.
**Impact of visualization**	

**Sharing/communication**<br>• HCP/Care team<br>• Public<br>• Caregivers	Any discussion of real or hypothetical use of visualizations to communicate with others, including the rationale, experiences, preferences, and outcomes of using visualizations in those contexts. Sub codes included common groups with whom service users reported using visualizations to communicate.
**Increased Self-awareness**<br>• Identifying patterns<br>• Seeing progress	Any description of how visualizations related to or impacted service users' self-awareness, usually regarding symptoms and triggers. Sub codes describe the use of visualizations to identify patterns (e.g., identify responses to a trigger, relating specific activities to symptoms) or seeing progress (e.g., seeing change over time or in response to an intervention)
**Validate current feelings/experiences**	Any description of how visualizations impacted services users' perception of the validity, acceptability, normality, or realness of their own symptoms
**Improve recall of past experiences**	Any description of how visualizations impact (actually or hypothetically) service users' ability to remember or recount historical symptoms or experiences
**Provide structure & organization**	Any description of how visualizations impact (actually or hypothetically) service users' ability to organize or structure their memories, symptom data, or approach to self-management
**Affecting self-image**	Any description of how visualizations impact (actually or hypothetically) service users' perception of themselves, their illness, or their abilities, either positively or negatively
**Engagement with RMT**	Any description of how visualizations impact (actually or hypothetically) engagement with remote monitoring technologies, either within a single session of using the RMT or over time.
**Enable proactive self-management**	Any description of how visualizations impact (actually or hypothetically) participants' ability or motivation to self-manage their conditions
**Moderators of visualization preferences/needs**	
**Health status**<br>• Disease severity<br>• Relapse<br>• Wellness	Any description of how health status impacts (actually or hypothetically) visualization design preferences. This could include impact of a user's condition or disease severity (ie, preferences in moderate vs. Severe disease), differing preferences before/during/after relapses or episodic events, or preferences in times of wellness vs. Those while feeling unwell.
**Personas**<br>• Data person<br>• Non data person<br>•	Any discussion of individuals being the 'type of person' who identifies with or prefers numbers, data, analytics, or qualitative reporting and visualization methods
**Experience with health monitoring**	Any discussion of how past experiences with health tracking or mHealth applications impact (actually or hypothetically) current design preferences

**Context/Intended use**	Any discussion of how context impacts (actually or hypothetically) current design preferences
**Customization**	
**Desire for customization**	Indication of a desire (or lack thereof) for customizing features of data visualizations
**Individual needs and interests**	Any discussion of customization, flexibility, personalization, or conflicting opinions due to individual experiences, priorities, and preferences
**Responsiveness to current status**	Any discussion of differing needs or preferences in specific situations (ie, when I'm depressed, do/don't show me…)
**Data reporting**	
**Translating feelings into objective information**	Any discussion of using visualizations to translate abstract experiences, feelings, or symptoms into objective data
**Identifying with data reporting mechanisms**	Any discussion of how a service user perceives their relationship with a data reporting mechanism
**Accuracy of quantitative vs. Qualitative data**	Any discussion on the perceived accuracy or validity of quantitative or qualitative data, within the context of visualization-based mechanisms
**Author response to service user feedback**	
**Author response to feedback**	Any description of design features or changes authors incorporated in response to service users' input or feedback

Illustrative Quotes

Table S5: Theme 1 - Desire for Data Visualization. Service users representing diverse conditions explicitly expressed desire for visualizing their personal data as part of an RMT application or service.

Descriptive theme	Illustrative quotes
**Desire for data visualization**	*"nearly all participants (14, 93.3%) would like to use a function that visualizes the Course of all monitored parameters."* [58] (service users with bipolar disorder)<br><br>*"they did not show particular interest in accessing data regarding their health status. However, when they were asked about their desired information from training sessions they indicated a preference for having access to step length, number of steps, and time of training."* [61] (service users with Parkinson's disease)
**Dissatisfaction with lack of visualization**	*"I kind of wanted to see my historical responses and that wasn't available."* [34] (service user quote, depression)<br><br>*"the fact that I am not able to look back at the information that I have put in the app is not a very motivating factor."* [42] (service user quote, narcolepsy)

Table S6: Theme 2 - Impact of visualizations on condition management

Descriptive theme	Illustrative quotes
**Enable proactive self-management**	*''it's probably made me more aware of . . . In that when I do think to look at the [true colours] graph I can say if my depression's dipping . . . Or going up even that I'll go right I'll go for a walk at lunchtime, you know so I suppose it gives me a little sense of control.''* [50] (service user quote, bipolar disorder)<br><br>*"I like how it gives you a up-to-the-minute look at the week to see how you are doing on your routine, which helps maintain the routine. Also, looking at the feedback you can figure out what might of happened to throw you off your routine"* [35] (service user quote, bipolar disorder)<br><br>*"what I saw [in the trial] is that it helped me keep on track. I try to keep track of the triggers [early warning Signs], and my history – and in that way it has helped me enormously. Previously, I went into periods where I encountered random mood swings, up and down, and I did not have any history [data] to relate to, so it kind of surprised me. But now I can actually follow how I'm doing – also back in time – and what caused it. It has really been great, and I think I have been able to keep track of myself."* [37] (author analysis of service user responses, bipolar disorder)

Table S7: Sub Theme 2.1 - Visualizations improve self-awareness and enable proactive self-management. Visualizations provided users with *structure and organization*, enabling them to more objectively *recall past experiences*. This increased objectivity allowed service users to *identify patterns* or triggers and *validate current feelings or experiences*. Heightened self-awareness enabled more *proactive self-management*, encouraging users to engage with RMT and *see progress over time*.

Descriptive theme	Illustrative quotes
**Provide structure and organization**	*"Still, respondents reported turning to external tracking via paper or technology when they need extra support, for instance, when thoughts get "scrambled" or their mind feels too "full" – sensations that are especially common during [bipolar disorder] episodes."* [46] (author analysis of service user responses, bipolar disorder)
**Improve recall of past experiences**	*"What I saw [in the trial] is that it helped me keep on track. I try to keep track of the triggers [early warning signs], and my history – and in that way it has helped me enormously. Previously, I went into periods where I encountered random mood swings, up and down, and I did not have any history [data] to relate to, so it kind of surprised me. But now I can actually follow how I'm doing – also back in time – and what caused it. It has really been great, and I think I have been able to keep track of myself."* [37] (service user quote, bipolar disorder)
**Validate current experiences**	*"I am looking for confirmation that I had similar symptoms in the past, because sometimes due to the nature of bipolar I feel like I can't trust the emotions I have at any given moment (or their possible triggers) and it is a relief to know that these are patterns."* [35] (service user quote, bipolar disorder)
**Increased self-awareness**	*"I found the most valuable tool to analyze my activities. It provides an understanding of which activities helps me, and which gives problems that I need to be aware of — or completely avoid."* [31] (service user quote, depression)
**Enable proactive self-management**	*"A majority of respondents described this awareness as an opportunity to become proactive about their condition, helping them make adjustments to preempt mood episode triggers and maintain stability or at least avoid severe episodes. Survey respondents also stated that the feedback provided by tracking keeps them accountable to themselves and that they find visual forms of feedback particularly helpful for identifying personal behavioral and emotional patterns and motivating positive lifestyle choices."* [46] (author analysis of service user responses, bipolar disorder)
**See progress over time**	*"I was amazed when I scrolled back through the android weeks to see how much my mood has stabilized since I started [medication]. The weeks themselves weren't as meaningful as the pattern over time."* [35] (service user quote, bipolar disorder)

Table S8: Sub Theme 2.2 - Visualizations enable more effective communication with care partners. Visualizations *improved recall of past experiences* and symptoms, helping both service users and their care partners *identify patterns*. Visualized data also provided *validation of the experiences* service users wished to convey to their care partners.

Descriptive theme	Illustrative quotes
**Sharing and communication**	*"Participants theorized that having access to objective data representing their symptoms, particularly in the graph form, might enable a shared understanding of their experiences, both with the care team ('maybe help themselves to understand a little bit better' [p227]) and potentially with the general public ('if it can help people in the future understand a little bit better about what we go through' [p227])."* [41] (author analysis of service user responses, schizophrenia)<br><br>*"Participants stated that these data also provide a starting point for dialogue with their physician, help them broach specific issues or points of concern, give them defensible evidence for discussions about treatment efficacy or medication adjustments, and, overall, have made them more 'active patients' who do not 'simply fill prescriptions'".* [46] (author analysis of service user responses, bipolar disorder)
**Improve recall of past experiences**	*''I think [the graph is] a really good thing to be looking at because I do find that if I'm not feeling well and then you don't get an appointment for a week it's so difficult to describe how you were feeling before and I think even with physical pain like as well as mood.''* [50] (service user quote, bipolar disorder)<br><br>*''If they could see at least I've been moving around and doing stuff during the week I think it would be useful for them. They have to take my word for it at the moment and although I won't lie there are some days I say I'm sorry I haven't got a clue what I did."*[50] (service user quote, bipolar disorder)
**Increase self-awareness, identify patterns**	*"I find that the reports succinctly provide my doctors with a more accurate picture over time than what I can recall at any given time. It also helps me to create a dialogue with my providers other than the fact that I don't feel well (mentally). It has also helped my providers to see symptoms and patterns that I wouldn't have thought to mention in short, 15 minute appointments."* [46] (service user quote, bipolar disorder)
**Validate current experiences**	*"If you were to answer the questions and go to the doctor and say "look, these are my results, you can see clearly there's a change, and these are my experiences," that would be substantial evidence for the doctor to then sit up and take note."* [41] (service user quote, schizophrenia)

Table S9: Sub Theme 2.3 - Visualizations drive engagement with RMT. Visualizations were perceived as engaging or even rewarding, providing reasons to return to the RMT over time.

Descriptive theme	Illustrative quotes
**Engagement with RMT**	*"Participants reported that the mood monitoring surveys and associated graphical feedback were a reason to return to the app and that it increased self-awareness of how their mood fluctuated over time and in relation to use of the intervention content."* [30] (author analysis of service user responses, depression)<br><br>*"Instead of just talking about whether logging has been occurring/its frequency, what about something like: 'your average wake time is 7:32 am based on five loggings this week.' you're rewarding the user with a bit of information that will likely motivate them to click and see more of their data."* [35] (service user quote, bipolar disorder)<br><br>*"To keep patients motivated to use the tool over a longer period, a personal visualization of recorded data is required."* [42] (author analysis of service user responses, narcolepsy)

Table S10: Theme 3 - Visualizations as data reporting mechanisms. Reactions to using visuals such as images, faces, and animations to report pain were mixed. Some service users were more able to *identify with visuals than traditional reporting mechanisms*, while others were not. Some found that visuals made it easier to *translate abstract feelings into objective data*, while others questioned the *accuracy of such a mechanism*.

Descriptive theme	Illustrative quotes
**Data reporting**	*"This is the first time I've been able to describe my pain to someone."* [39] (service user quote, chronic pain)
**Translating feelings into objective information**	*"Such individuals also thought using faces to express their pain felt more natural and intuitive, e.g.:*<br>*'with the numbers, my brain needs to actually think more about the body part and the pain sensation explicitly and somehow try to map that onto a number. Face is a different translation channel that goes through emotion and feeling, though the underlying component is still my pain.'"* [40] (author analysis of service user responses, chronic pain)
**Identifying with data reporting mechanisms**	*"Those who valued reporting with faces were able to connect with the people and expressions displayed (e.g., 'I can definitely imagine feeling the way this person looks' – p2; 'I can kind of imagine him [safe] feeling what I feel, feeling more than just the sterile measure' – p3)."* [40] (author analysis of service user responses, chronic pain)<br><br>*"Two participants suggested that using photos of their own faces to report pain would be more useful instead."* [40](author analysis of service user responses, chronic pain)
**Accuracy of quantitative vs. Qualitative data**	*"Several participants doubted the accuracy of facial expression based measures (e.g., 'I don't like coordinating pain to facial expressions because I just can't believe it's accurate' – p4)"* [40] (author analysis of service user responses, chronic pain)

Table S11: Theme 4 – Visualization design considerations. Users shared diverse perspectives on visualization design preferences. However, they frequently emphasized the importance of *contextual detail, timeliness, and the ability to customize visualizations* according individual needs.

Descriptive theme	Illustrative quotes
**Visualization design**	*"Similar to other personal health systems that use fishes and flowers as metaphors, we were looking for an appropriate metaphor for bipolar disorder. Many attempts were tried, including using metaphors like a scale, an equalizer, a river, a volcano, a dart board, and a radar, but we always had the case that some patients preferred one visualization, and others hated it"* [37] (author analysis of service user responses, bipolar disorder)
**Format**	One participant *"gravitated towards more interconnectivity of raw data from Medicare and data from multiple apps aggregated in one graph"* [45] (author analysis of service user responses, mental health condition)<br><br>*"Screens used a circular progress bar that also incorporates color coding to provide the user with unambiguous feedback regarding their progress"*[61] (author description of the visualization, Parkinson's disease)<br><br>*"Mood was believed to be conveyed through colour; therefore, the app was given a function that Enabled the service user to assign different colours to their mood. Of the preferred ways that mood Could be represented was a bar graph with smiley faces. One service user liked the use of a 'mood Cloud' to convey changes in mood."*[51] (author analysis of service user responses, mixed mental health conditions)
**Context and annotation**	*"I would like to click [points on the daily mood graph] and see what I did on this day, since I was so well." [31] (service user quote, depression)*<br><br>*"I kinda wish I could put a little note in and be like, 'this is why I put this number.' I think, yeah. I guess that's actually - it would have been really nice to have some sort of journaly type feature where I could make notes like that"* [34] (service user quote, depression)
**Timeliness**	*"Comments mostly differed regarding the frequency of [feedback via data visualizations], again with a great range from daily feedback to one response per month:*<br>• *'yes, one feedback per month. Or maybe every two weeks. In the case of warning signals also more frequently.'*<br>• *'yes a short feedback every two days or every day.'*<br>• *'yes absolutely. Once a week would be good or every two weeks. Not every day though.'"*<br>[58] (author analysis of service user responses, bipolar disorder)
**Customization**	*"Survey respondents' approaches for tracking multiple indicators vary. Participants were divided between keeping separate journals or tools, each dedicated to chronicling a particular indicator, or tracking all items with a single chart or application. Sometimes, elaborate tracking setups are reported as necessary to accommodate such tracking habits in ways technologies do not currently support"* [46] (author analysis of service user responses, bipolar disorder)

Table S12: Sub theme 4.4 – Moderators of user needs and design preferences. Moderators such as *health status, data literacy, and experience with RMT* can affect design preferences and necessitate flexibility.

Theme	Illustrative quotes
**Health status**	*"The need for goal-setting and proper feedback was deeply emphasized... Being able to understand when progress is being achieved was considered key as the subjective experiences differed from what [the service users] actually accomplished" [47]* (author analysis of service user responses, multiple sclerosis)<br><br>*"Some patients expressed concern that the red color used to display high symptom scores may be disturbing to patients who already knew they were doing poorly."* [32] (author analysis of service user responses, depression)
**Data literacy and "data people"**	*"I think I'm the sort of person that I like to see the data around whatever problem I've got, just to help me understand it and monitor it. So I'm always really interested in seeing the statistic."* [45] (service user quote, mental health condition)<br><br>*"One participant described a sense that numbers might be better suited to scientific applications but are too sanitary and impersonal to reflect non-objective feelings like pain for all end-users: 'numbers might make more sense for a health professional, but I'm not sure they do for me. No-numbers is somehow more descriptive'"* [40] (author analysis of service user responses: chronic pain)
**Experience with self-monitoring**	*"Once individuals have been managing their [bipolar disorder] for years, they describe gaining a sense of control that stems from better understanding their personal condition and how their routine affects it. They explain that it eventually becomes possible to rely on mental tracking and less on overt tracking strategies, which are used more as a fallback when something out of the ordinary happens or in times of severe emotional distress."* [46] (author analysis of service user responses, bipolar disorder)
